# Drug Repurposing in Oncology: A Systematic Review of Randomized Controlled Clinical Trials

**DOI:** 10.3390/cancers15112972

**Published:** 2023-05-30

**Authors:** Ignatios Ioakeim-Skoufa, Natalia Tobajas-Ramos, Enrica Menditto, Mercedes Aza-Pascual-Salcedo, Antonio Gimeno-Miguel, Valentina Orlando, Francisca González-Rubio, Ana Fanlo-Villacampa, Carmen Lasala-Aza, Ewelina Ostasz, Jorge Vicente-Romero

**Affiliations:** 1 WHO Collaborating Centre for Drug Statistics Methodology, Department of Drug Statistics, Division of Health Data and Digitalisation, Norwegian Institute of Public Health, NO-0213 Oslo, Norway; 2 EpiChron Research Group, Aragon Health Research Institute (IIS Aragón), Miguel Servet University Hospital, ES-50009 Zaragoza, Spain; 3 Research Network on Chronicity, Primary Care, and Health Promotion (RICAPPS), Institute of Health Carlos III (ISCIII), ES-28029 Madrid, Spain; 4 Drug Utilization Work Group, Spanish Society of Family and Community Medicine (semFYC), ES-08009 Barcelona, Spain; 5 Department of Pharmacology, Physiology, and Legal and Forensic Medicine, Faculty of Medicine, University of Zaragoza, ES-50009 Zaragoza, Spain; 6 Centro Interdipartimentale di Ricerca in Farmacoeconomia e Farmacoutilizzazione (CIRFF), Center of Drug Utilization and Pharmacoeconomics, Department of Pharmacy, University of Naples Federico II, IT-80131 Naples, Italy; 7 Primary Care Pharmacy Service Zaragoza III, Aragon Health Service (SALUD), ES-50017 Zaragoza, Spain; 8 Pharmacy Service, Virgen de la Victoria University Hospital, ES-29010 Malaga, Spain; 9 Rehabilitation Centre Vikersund Bad AS, NO-3370 Vikersund, Norway

**Keywords:** drug repositioning, antineoplastic agents, medical oncology, mebendazole, metformin, propranolol, etodolac, imatinib, leuprolide, multimorbidity

## Abstract

**Simple Summary:**

Exploring the possibility of using well-known marketed drugs in new therapeutic indications, commonly known as drug repurposing, offers certain advantages over discovering new substances for medicinal use; it saves time and costs and reduces risks as the safety profile is, in many cases, well-established. This approach has grasped the interest of scientists for one of the most lethal conditions worldwide—cancer. Several preclinical and observational studies showed that various drugs may benefit oncological patients. Placebo- or no intervention-controlled clinical trials can offer evidence regarding the efficacy of a drug in a particular therapeutic indication. This systematic review summarizes randomized controlled clinical trials that evaluate drug repurposing possibilities in cancer for drugs that are currently authorized for non-oncological indications.

**Abstract:**

Quality pharmacological treatment can improve survival in many types of cancer. Drug repurposing offers advantages in comparison with traditional drug development procedures, reducing time and risk. This systematic review identified the most recent randomized controlled clinical trials that focus on drug repurposing in oncology. We found that only a few clinical trials were placebo-controlled or standard-of-care-alone-controlled. Metformin has been studied for potential use in various types of cancer, including prostate, lung, and pancreatic cancer. Other studies assessed the possible use of the antiparasitic agent mebendazole in colorectal cancer and of propranolol in multiple myeloma or, when combined with etodolac, in breast cancer. We were able to identify trials that study the potential use of known antineoplastics in other non-oncological conditions, such as imatinib for severe coronavirus disease in 2019 or a study protocol aiming to assess the possible repurposing of leuprolide for Alzheimer’s disease. Major limitations of these clinical trials were the small sample size, the high clinical heterogeneity of the participants regarding the stage of the neoplastic disease, and the lack of accounting for multimorbidity and other baseline clinical characteristics. Drug repurposing possibilities in oncology must be carefully examined with well-designed trials, considering factors that could influence prognosis.

## 1. Introduction

Drug repurposing, also called drug repositioning, is the process of discovering new uses outside the scope of the original medical indication for existing drugs [1]. It offers important advantages in comparison with traditional drug development procedures. While de novo drug discovery and development may require a 10- to 17-year procedure with a low overall probability of success, drug repurposing may reduce time and risk, as several questions and issues regarding drug discovery and development have been previously addressed [1,2,3]. In addition, pharmacovigilance systems continuously collect data regarding the safety profile of the marketed drugs in the real-world setting; this is vital considering multimorbidity (i.e., the coexistence of multiple chronic conditions) and polypharmacy in an aging population with, consequently, well-established and yet-to-discover drug-drug, drug-disease, and disease-disease interactions and relationships.

After the identification of a potential new indication and compound identification and acquisition, drug development may start in preclinical Phase I or Phase II stages, saving time, risk, and costs. In drug repurposing, one of the major objectives during drug development is to study the efficacy of the drug in the new indication under investigation [1]. Randomized, controlled clinical trials can offer high-quality evidence in this regard. Placebo-controlled or no intervention-controlled studies can generate evidence on the efficacy of the drug in the new indication, assess risk/benefit, and compare with well-established standards of care. Dose-escalation studies help identify the optimal dose for treatment.

Chronic diseases and multimorbidity challenge public health systems worldwide and constitute a global health research priority [4]. Approximately seven in ten deaths are attributed to chronic conditions; cardiovascular diseases, cancers, chronic respiratory diseases, and diabetes account for over 80% of all premature deaths related to chronic diseases [5,6]. The World Health Organization’s global action plan for the prevention and control of noncommunicable diseases 2013–2020, extended till 2030, includes an important reduction in the risk of premature death and an 80% availability of the affordable basic technologies and essential medicines required to treat these major chronic diseases [7,8]. In this systematic review, we focus on cancer, the second leading cause of death globally, accounting for one in six deaths [9].

Cancer is one of the most lethal diseases, with a significantly high mortality rate. Prevention mechanisms and research on the human genome offer the possibility of improving cancer diagnosis and treatment [10,11]. Since the 1950s, 5-fluorouracil has been increasingly used and has remained the backbone of most chemotherapy regimens. Several methods related to the function of non-coding transcripts in the modulation of cells can help in the therapeutic effect of 5-fluorouracil [12]. Currently, there are many therapeutic options, including surgery, chemotherapy, radiation therapy, immunotherapy, and biologic agents [13]. In recent years, many studies have been carried out to find new therapeutic alternatives, including repositioning [13].

Accessible early detection and quality treatment can improve survival for many types of cancer [9]. Significant work is in progress for novel, efficient strategies in cancer treatment; a promising approach is drug repurposing [14]. We aimed to perform a systematic review in MEDLINE to identify randomized placebo- or no intervention-controlled clinical trials that evaluate drug repurposing possibilities in cancer for marketed drugs that are currently authorized for non-oncological indications. A secondary objective is to identify trials that aim to study the potential use of antineoplastic agents in other non-oncological conditions.

## 2. Materials and Methods

We conducted a systematic review of the peer-reviewed literature in MEDLINE, following the preferred reporting items for systematic reviews and meta-analyses (PRISMA) statement guidelines (see Supplementary Material File S1) [15]. The search strategy combined algorithms for drug repurposing and neoplasms, as shown in Table 1. MEDLINE was selected as a data source as it comprises more than 35 million citations for biomedical literature, covering most of the research literature in the field.

**Figure 1:**
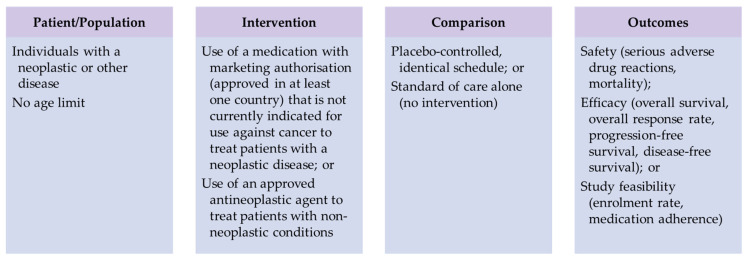
Application of the Patient/Population, Intervention, Comparison, and Outcomes (PICO) model to assess the suitability of the identified articles for inclusion in the systematic review.

In this study, we included articles that met all the following criteria: (i) the study was a randomized controlled clinical trial (RCT); (ii) the full text is available; (iii) the paper is in English or Spanish; and (iv) it answers the research question. To properly address this last criterion, we applied the Patient/Population, Intervention, Comparison, and Outcomes (PICO) model [16], as shown in Figure 1.

Information from study protocols was also recorded to include RCTs that are in progress. We performed the literature review on 20 November 2022. Three researchers (I.I.-S., N.T.-R., and J.V.-R.) screened titles, abstracts, and full text when considered necessary in pairs, following a double-blind method, to exclude irrelevant articles. When there was disagreement, a final decision was made by consensus. Relevant articles that were cited in the reference list of the included studies and met all the inclusion criteria were also screened for inclusion in the systematic review.

We extracted data regarding the year of publication, country, study period, clinical trial phase, masking, potential new indication in investigation, drug, route of administration, the aim of the study, time frame, target population, age of patients, number of enrolled individuals, intervention group, control group, loss to follow-up, mortality, outcome, main findings, serious adverse drug reactions, authors’ conclusions, limitations, and funding, amongst others. We recorded if multimorbidity was considered. The quality of evidence was assessed following the grading of recommendations assessment, development, and evaluation (GRADE) system [17,18]; a detailed report of the assessment is presented in the Supplementary Material Table S1.

## 3. Results

### 3.1. Literature Search Results

**Figure 2:**
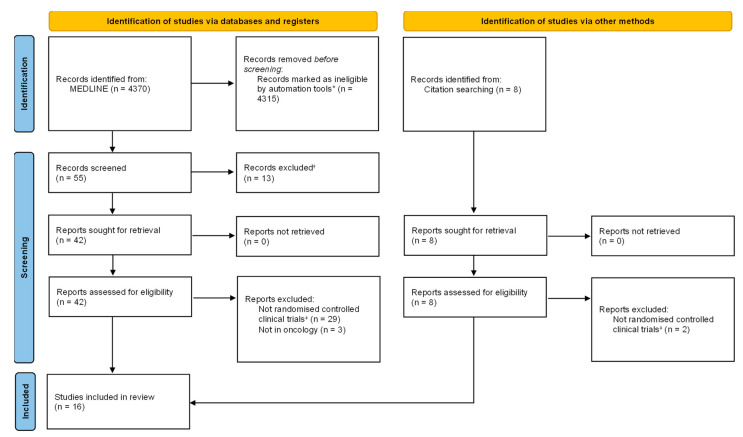
PRISMA 2020 flow diagram. * PubMed filters; studies eligible for screening: Clinical Trial, Clinical Trial, Phase I, Clinical Trial, Phase II, Clinical Trial, Phase III, Clinical Trial, Phase IV, Clinical Trial Protocol, Controlled Clinical Trial, Pragmatic Clinical Trial, Randomized Controlled Trial). ^ⴕ^ Human-review, by consensus (records that were not on drug repurposing in oncology were excluded). ^‡^ To be included in the study, control group should be placebo or no intervention (standard of care alone). From Page, M.J., et al. [15], for more information, visit: http://www.prisma-statement.org/ (accessed on 24 February 2023).

The literature search in MEDLINE gave 55 potentially relevant publications (Figure 2). After the screening, we excluded 13 publications that were not investigating the repurposing of an approved antineoplastic agent for a new non-oncological indication or the repurposing of a drug used in non-oncological conditions for the pharmacological treatment of cancer. We assessed for eligibility the remaining 42 articles plus eight new articles identified as potentially relevant records via citation searching. Finally, 16 articles were included in the review; eight of them were study protocols.

### 3.2. Excluded Articles

Many of the clinical trials excluded were not randomized controlled trials. Most of them were dose-escalation studies without a placebo- or standard-of-care-alone-control group. Examples were trials that studied the potential use of the antiparasitic agent mebendazole [19,20,21], various renin-angiotensin system modulators [22], or disulfiram [23], for gliomas; the antiprotozoal pyrimethamine [24], or the immunosuppressant leflunomide [25], for hematological cancers; and various other drugs for gynecological cancer [26], non-small cell lung cancer [27], prostate cancer [28,29], and head and neck cancers [30]. Some trials used historical data to compare, for example, a trial that studied the potential to use metformin for non-small cell lung cancer [31]. One clinical trial studied the potential to use valproic acid for various types of cancer in the pediatric population [32]. There were also trials aiming to study the potential repurposing of antineoplastic agents for other non-oncological conditions, for example, aldesleukin for type 1 diabetes mellitus [33] and the study protocol for the potential use of bosutinib in amyotrophic lateral sclerosis [34]. In general, most of these studies reported a good safety profile for the drug under investigation. Results regarding efficacy are very heterogeneous among the studies, and further research is needed in most of the cases.

### 3.3. Drugs in Investigation for Repurposing and Use in Oncological Conditions

Our study identified seven randomized clinical trials that evaluated the potential use of drugs currently used in non-neoplastic diseases for the pharmacological treatment of various types of cancer (Table 2). These trials studied the use of mebendazole for metastatic colorectal cancer [35], metformin for locally advanced and metastatic hormone-sensitive prostate cancer [36], metformin for advanced or metastatic non-squamous non-small cell lung cancer [37], propranolol in autologous hematopoietic stem cell transplantation for multiple myeloma [38], low-weight heparin for esophageal squamous cell carcinoma [39], propranolol and etodolac for breast cancer stages I–III [40], and metformin for metastatic or unresectable locally advanced pancreatic adenocarcinoma [41]. Most of the clinical trials aimed to study safety, efficacy, and feasibility. The most common main outcomes were overall survival (OS), progression-free survival (PFS), and the occurrence of adverse drug reactions (ADRs). All these trials enrolled only adults. Common limitations were the small sample size (most of them had less than 100 participants) and the high clinical heterogeneity of the study population. None of the trials considered multimorbidity. In general, overall survival was not significantly improved in the intervention group compared to the control group. Some studies reported higher progression-free survival in the intervention group. A detailed description of all randomized clinical trials, including information regarding safety and efficacy, is presented in Supplementary Table S1.

Our literature search identified five study protocols (Table 3) [42,43,44,45,46]. These trials aim to study safety, efficacy, feasibility, medication adherence, and health-related quality of life. Drugs under investigation include meclofenamate, sodium valproate, propranolol, etodolac, atorvastatin, and disulfiram.

**Table 2:** Characteristics of included studies that presented results in the systematic review.

Author (Year)	Country	Drug	Aim	Time Frame	Potential New Indication in Investigation	Main Findings	Author’s Conclusions	Limitations	Quality of Evidence (GRADE)
**Drugs under investigation for use in oncological conditions**									
Hegazy S.K. et al. (2022) [35]	Egypt	Mebendazole	Anti-tumor activity and safety	12 months (mean)	Metastatic colorectal cancer (stage 4)	ORR improved 12 weeks after treatment but not significantly after 12 months; one-year OS did not significantly improve	Mebendazole was well tolerated and showed anti-tumor activity	Small sample size, high drop-out, molecular tumor characteristics not considered, lack of intention-to-treat analysis	Moderate ⨁⨁⨁◯
Alghandour R. et al. (2021) [36]	Egypt	Metformin	Efficacy and safety	22 months (mean)	Hormone-sensitive prostate cancer	The median CRPC-free survival was higher in the metformin group (*p* = 0.01). In patients with metastatic disease, there was no difference (*p* = 0.15)	Patients with high-risk localized disease, regional lymph node metastases, and those with metastatic low tumor volume disease seem to derive most of the benefit	Control group was standard of care and not placebo-controlled; it was a heterogenous population with heterogenous interventions (as standards of care)	Moderate ⨁⨁⨁◯
Marrone K.A. et al. (2018) [37]	USA	Metformin	Efficacy and safety	12 months	Advanced or metastatic NSCLC	There was a significant benefit in PFS with the use of metformin (*p* = 0.024), but OS was not significantly different	Metformin is a well-tolerated drug that, in addition to standard chemotherapy, can improve progression free survival	Due to the small sample size, the study was stopped because of changes in practice patterns for treatment, a lack of correlative analyses, and open-label	Moderate ⨁⨁⨁◯
Knight J.M. et al. (2018) [38]	USA	Propranolol	Efficacy, safety, and feasibility	100 days	Multiple myeloma	Enrollment rate: 16%; no serious ADRs were reported; MA: 94%	It is feasible to recruit and treat multiple myeloma patients with propranolol during HCT, with the greatest obstacle being other competing oncology trials	Small sample size, open-label	Moderate ⨁⨁⨁◯
Taghizadeh Kermani A. et al. (2018) [39]	Iran	Enoxaparin	Efficacy and safety	7 months	Oesophageal squamous cell carcinoma	Integration of enoxaparin into the chemoradiation protocol is safe and tolerable. Higher probability of neutropenia	The clinical and pathological response of squamous cell carcinoma to neoadjuvant chemoradiation was improved by the addition of enoxaparin (the difference was not significant)	Small sample size and no information about anti-Xa levels	Moderate ⨁⨁⨁◯
Shaashua L. et al. (2017) [40]	Israel	Propranolol; etodolac	Efficacy and safety	16 days (mean)	Primary operable breast cancer stages I–III	Decreased epithelial-to-mesenchymal transition, reduced activity of pro-metastatic/pro-inflammatory transcription factors, and decreased tumor-infiltrating monocytes while increasing tumor-infiltrating B cells	Perioperative inhibition of COX-2 and b-adrenergic signaling provides a safe and effective strategy for inhibiting multiple cellular and molecular pathways related to metastasis and disease recurrence in early-stage breast cancer	No information about long-term clinical outcomes	High ⨁⨁⨁⨁
Kordes S. et al. (2015) [41]	Netherlands	Metformin	Efficacy and safety	6 months	Pancreatic adenocarcinoma	OS at 6 months was higher in the placebo group (*p* = 0.41). Median OS was higher in the placebo group (hazard ratio 1.056 [95% CI 0.72–1.55])	There is no advantage to the addition of metformin to erlotinib and gemcitabine in the treatment of advanced pancreatic cancer	No information on tumor biomarkers; high patient heterogeneity; and open-label	Moderate ⨁⨁⨁◯
**Antineoplastic agents under investigation for use in non-oncological conditions**									
Aman J. et al. (2021) [47]	Netherlands	Imatinib	Efficacy and safety	28 days	COVID-19 with hypoxic respiratory failure	There was no significant differences between the intervention and control groups regarding the time to discontinuation of supplemental oxygen and mechanical ventilation (HR 1.07, 95% CI 0.62–1.84; *p* = 0.82; adjusted for baseline characteristics)	Imatinib did not reduce the time to discontinuation of ventilation and supplemental oxygen for more than 48 consecutive hours in patients with COVID-19 requiring supplemental oxygen	Loss of follow-up (partly due to hospital relocations during the pandemics), imbalances in sex baseline clinical characteristics (comorbidities), and the treatment period of ten days were based on earlier observations and might need to be reconsidered	High ⨁⨁⨁⨁

Abbreviations: ADRs, adverse drug reactions; CI, confidence interval; COVID-19, coronavirus disease 2019; COX, cyclooxygenase; CRPC, castration-resistant prostate cancer; HCT, hematopoietic cell transplantation; HR, hazard ratio; MA, medication adherence; MGMT, O-6-methylguanine-DNA methyltransferase; NSCLC, non-small-cell lung cancer; ORR, overall response rate; OS, overall survival; PFS, progression-free survival; quality of evidence GRADE rating, ⨁◯◯◯ very low, ⨁⨁◯◯ low, ⨁⨁⨁◯ moderate, ⨁⨁⨁⨁ high.

**Table 3:** Characteristics of included protocols in the systematic review.

Author (Year)	Country	Drug	Aim	Time Frame	Potential New Indication in Investigation	Main Outcomes
**Drugs under investigation for use in oncological conditions**						
Zeyen T. et al. (2022) [42]	Germany	Meclofenamate	Efficacy, safety, tolerability, and quality of life	6 months	Progressive MGMT-methylated glioblastoma	OS, PFS, ADRs, and QoL
McCarthy C. et al. (2021) [43]	UK	Sodium valproate	Clinical activity, mechanism of action, and study feasibility	6 months	High-risk oral epithelial dysplasia	Changes in lesion size, changes in histological grade, and loss of heterozygosity
Hüttner F.J. et al. (2020) [44]	Germany	Propranolol; etodolac	Safety, feasibility, and early parameters of efficacy	24 months	Elective pancreatic head resection	Serious ADRs, post-operative mortality, pancreas-associated morbidity, MA, OS, DFS, and rates of local and distant recurrence
Polster S.P. et al. (2019) [45]	USA	Atorvastatin	Efficacy	24 months	Cavernous angiomas	Change in QSM per year using intention-to-treat analysis, vascular permeability, andADRs
Jakola A.S. et al. (2018) [46]	Norway, Sweden	Disulfiram	Efficacy, safety, and health-related quality of life	24 months	Recurrent glioblastoma	Six-month survival (primary endpoint), OS, PFS, safety, and health-related QoL
**Antineoplastic agents under investigation for use in non-oncological conditions**						
Atmowihardjo L. et al. (2022) [48]	Netherlands	Imatinib mesylate	Efficacy, safety, and tolerability	28 days	COVID-19 with acute distress respiratory syndrome	Change in Extravascular Lung Water Index between baseline (day 1) and day 4, SOFA score, 28-day mortality, ADRs
Butler T. et al. (2021) [49]	USA	Leuprolide	Efficacy	52 weeks	Alzheimer’s disease	Change in cognition from baseline to post-treatment as measured by the ADAS-Cog
Emadi A. et al. (2020) [50]	USA	Imatinib	Efficacy, safety, tolerability, and pharmacokinetics	60 days	COVID-19	Proportion of patients with a two-point improvement at day 14 from baseline using an 8-category ordinal scale; all-cause mortality at day 28 and at day 60

Abbreviations: ADAS-Cog, Alzheimer’s Disease Assessment Scale–Cognitive subscale; ADRs, adverse drug reactions; COVID-19, coronavirus disease 2019; DFS, disease-free survival; MA, medication adherence; MGMT, O-6-methylguanine-DNA methyltransferase; OS, overall survival; PFS, progression-free survival; QoL, quality of life; QSM, quantitative susceptibility mapping; SOFA, sequential organ failure assessment.

### 3.4. Antineoplastics in Investigation for Repurposing and Use in Non-Oncological Conditions

We identified one randomized placebo-controlled clinical trial studying the potential repurposing of an antineoplastic agent for use in a non-oncological condition (Table 2). The drug under investigation was imatinib, a BCR-ABL tyrosine kinase inhibitor, and the potential new indication was the treatment of patients with severe acute respiratory syndrome coronavirus 2 (SARS-CoV-2) infection and hypoxic respiratory failure [47]. Multimorbidity was taken into consideration in this clinical trial. The main outcome of the study was the time to discontinue ventilation and supplemental oxygen for more than 48 consecutive hours while alive; the study reported no significant differences between the intervention and the control group.

We identified three study protocols (Table 3) [48,49,50]. Two trials aim to study the potential use of imatinib in coronavirus disease 2019 (COVID-19) [48,50]. The other clinical trial investigates the possible repurposing of leuprolide, a gonadotropin-releasing hormone analogue, for the treatment of patients with Alzheimer’s disease [49].

## 4. Discussion

Our study revealed that few clinical studies on drug repurposing in oncology were randomized placebo-controlled or standard-of-care-alone-controlled trials. These studies mainly assess the efficacy and safety of the drug in conditions outside the scope of the authorized indications. This objective was commonly assessed through the overall response rate, overall survival, progression-free survival, disease-free survival, and the notification of drug-related side effects and adverse reactions. Among the most common limitations of the identified studies were the small sample size (most of them had less than 100 participants), the high clinical heterogeneity of the participants, and the lack of accounting for multimorbidity and other baseline characteristics.

Metformin has been studied for potential use in some types of cancer in combination with standard treatment. A blinded RCT with 124 enrolled patients with locally advanced and metastatic hormone-sensitive prostate cancer showed that metformin, in combination with standard of care, prolonged castration-resistant prostate cancer-free survival by nine months in comparison with the group treated with standard of care alone (29 vs. 20 months; *p* = 0.01) [36]. The difference was particularly observed in localized disease, whereas in advanced or metastatic cases it was found to be not statistically significant. There were no differences regarding PSA levels or overall survival between the two groups. Concurrent diabetes mellitus (approximately present in 20% of the study population; both metformin- and control-group) was not identified as a predictor of shorter castration-resistant prostate cancer-free survival. Another open-label clinical trial with 25 enrolled non-diabetic patients with chemotherapy-naive advanced or metastatic non-squamous non-small-cell lung cancer (NSCLC) showed that metformin has the potential to improve progression-free survival within three months when combined with chemotherapy compared with chemotherapy alone (9.6 vs. 6.7 months, *p* = 0.024), without significant difference regarding overall survival [37]. However, an open-label RCT with 121 patients with metastatic or unresectable locally advanced pancreatic adenocarcinoma reported lower overall survival when metformin was added compared with placebo to gemcitabine and erlotinib, although the difference was not statistically significant (6.8 vs. 7.6 months, *p* = 0.78) [41]. The authors reported similar findings regarding progression-free survival in favor of the placebo group, although differences were not statistically significant (4.1 vs. 5.4 months, *p* = 0.44). The small sample size and the high clinical heterogeneity of the enrolled patients were among the most important limitations of the clinical trials.

Epidemiologic studies have suggested that type 2 diabetes mellitus, insulin resistance, and hyperinsulinemia might be associated with a higher risk of cancer [51]. Many retrospective observational studies reported findings that call for an in-depth study of the possible beneficial use of metformin in some types of cancer [52,53,54,55,56,57,58,59,60,61,62], boosting interest in studying the interlinking metabolic pathways between diabetes and cancer and the role, if any, of metformin. However, there are discrepancies in the literature and various limitations and questions that should be properly addressed, mostly regarding several confounding factors. For example, comorbid diabetes (considering year of onset, treatment, and complications), other comorbidities, clinical status, and concomitant medication, medication adherence, potentially drug-drug and drug-disease interactions, current clinical management, patient’s preferences and socioeconomic status, lifestyle factors, and many other variables could influence prognosis. A recent meta-analysis showed that metformin was found to improve patient outcomes in patients with head and neck cancer in studies that did not adjust for comorbidities; in studies that adjusted for comorbidities, no significant improvement was found [63]. The potential of repurposing a widely used and safe drug, a drug that is included in the World Health Organization’s model List of Essential Medicines [64], must be carefully examined with well-designed RCTs. It is also essential to evaluate the posology and administration regimens in order to achieve optimal delivery to the tumor with acceptable tolerability and antineoplastic activity [65]. It is important to keep in mind other potential uses of metformin for many other indications, currently, in clinical research—highlighting even more the necessity to consider comorbidity in the clinical trials on drug repurposing—describing metformin as the drug of the future fighting a multitude of diseases [66]; although the exact mechanism is unknown, some of these findings may be attributed to its insulin-sensitizing and anti-hyperglycemic effects [67].

Mebendazole is a broad-spectrum antihelminthic that has been in use for more than five decades [68]. The first observations of a potential anticancer effect of mebendazole were reported in 2002 [69,70]; they were preclinical studies in lung cancer. Among the antitumoral effects suggested were angiogenesis inhibition [69] and tubulin depolymerization [70]. Since then, other anticancer effects have been attributed to mebendazole [71,72,73,74], including inhibition of the Hedgehog signaling pathway [75], induction of apoptosis and cytotoxicity [76], inhibition of kinases [77], induction of a pro-inflammatory (M1) phenotype of monocytoid cells [78], and sensitization to chemotherapy and radiotherapy [79]. Various preclinical studies, in vitro and in vivo, reported anticancer activity [72] in a wide range of cancer types, such as non-small cell lung cancer [69,70], glioblastoma [80,81], melanoma [82,83], acute myeloid leukemia [84], ovarian cancer [85], and colorectal cancer [86]. Two case reports reported favorable outcomes: long-term tumor control in a patient with metastatic adrenocortical carcinoma [87] and nearly complete remission of the metastases in the lungs and lymph nodes in a patient with metastatic colon cancer [88]. At the moment, information from clinical trials is limited. We identified one randomized controlled clinical trial with 64 patients with metastatic colorectal cancer (stage 4) in treatment with bevacizumab and FOLFOX4 [35]. Twelve weeks after treatment with mebendazole (in addition to standard baseline therapy), it was observed that the overall response rate improved and vascular endothelial growth factor declined in comparison with the control group (only baseline treatment); however, after one year, differences were not significant. Among the most common adverse events and drug reactions were gastrointestinal (abdominal pain and diarrhea) and biochemical alterations (aspartate transaminase, alanine transaminase, serum creatinine, and estimated creatinine clearance). A phase 1 study in 11 patients with relapsed or recurrent high-grade gliomas aimed to identify the maximum tolerable dose of mebendazole in combination with other therapeutic approaches (lomustine, temozolomide, or re-radiation plus temozolomide) [19]. This escalation study (dose range 100–1600 mg three times daily) suggested that the recommended phase 2 dose of mebendazole is 1600 mg three times daily when combined with temozolomide or re-radiation plus temozolomide and 800 mg when combined with lomustine. In a phase 2 study, the authors reported that using these recommended doses, these combinations of mebendazole did not reach the pre-defined benchmark of 55% overall survival at nine months [20]. Regarding safety profile, another dose-escalation phase 1 study reported long-term safety and acceptable toxicity with doses up to 200 mg/kg [21].

Propranolol is the first successfully developed beta-blocker [89,90]. It is mainly used in hypertension, cardiac disease, and other conditions, including infantile hemangiomas [91,92,93]. Various studies during the last 25 years showed that beta-blockers may have anti-proliferative properties and inhibit metastasis in a variety of cancers [94,95,96,97,98,99,100,101,102,103], such as lung [94], colorectal [100], prostate [99], and ovarian cancer [101]. Other studies reported better clinical outcomes in multiple myeloma [98,102,103,104]. Better prognosis in multiple myeloma, especially when treated with hemopoietic stem cell transplantation, may be partly attributed to the blockade of the sympathetic nervous system in the bone marrow niche induced by b-blockers [102,104,105,106]. A clinical trial reported that, although challenging, it is feasible to recruit and treat multiple myeloma patients with propranolol during hemopoietic stem cell transplantation [38]. The same study reported a good safety profile with the use of propranolol, with the most common adverse reactions being hypotension, dizziness, maculopapular rash, hypokalemia, hypertension, and chest pain. There is a need for further clinical studies, primarily randomized controlled clinical trials, that also consider molecular tumor characteristics and the baseline clinical profile of the patients.

Various studies suggest that combining b-blockers and cyclooxygenase-2 (COX-2) inhibitors may be beneficial in some types of cancer, suppressing cancer progression [107,108]. The effect of COX-2 inhibitors may be attributed to different mechanisms of action [107], including apoptosis [109] and an anti-angiogenic action [110]. Furthermore, it has been reported that some cancers secrete prostaglandins to escape destruction, such as renal cell carcinoma [111]. Perioperative b-blockers in combination with COX-2 inhibitors may improve immune competence and reduce the risk of metastasis [107,108]. A randomized clinical trial in patients with primary operable breast cancer reported a decreased epithelial-to-mesenchymal transition, reduced activity of prometastatic/proinflammatory transcription factors, and decreased tumor-infiltrating monocytes while increasing tumor-infiltrating B cells in patients who received propranolol and etodolac [40]. Regarding the safety profile, no severe or moderate adverse events were observed, with nausea among the most common. Another clinical trial is in progress in patients with resectable carcinoma of the pancreatic head planned for pancreatoduodenectomy, aiming to study serious adverse drug reactions, post-operative mortality, pancreas-associated morbidity, medication adherence, overall and disease-free survival, and rates of local and distant recurrence [44].

There is a lot of discussion regarding interactions and relations, especially common etiopathogenic mechanisms, between cardiovascular and oncological conditions [112]. Revealing common pathophysiological factors generates hypotheses for repurposing well-known cardiovascular drugs for potential use in oncological diseases, amongst them b-blockers (as previously discussed), angiotensin receptor antagonists, statins, and low molecular weight heparins. Our review identified a protocol for a randomized controlled clinical trial studying the efficacy and potential use of atorvastatin in patients with cavernous angiomas [45]. Many studies reported a potential benefit of heparin in cancer patients, suggesting that low-weight heparins may have direct anti-metastatic effects above their anticoagulation properties [113,114,115,116,117]. Studies include various types of cancer, such as multiple myeloma, pancreatic cancer, prostate cancer, and hepatocellular carcinoma [118,119]. A randomized controlled clinical trial in patients with esophageal squamous cell carcinoma reported that integration of enoxaparin into the chemoradiation protocol is safe and tolerable; however, a higher probability of neutropenia was observed in patients treated with enoxaparin [39]. There are conflicting results in the literature regarding the overall benefit of heparin in oncology [118]. It is crucial to conduct further research to fully understand the biological mechanisms, the clear benefit, and the risks of using heparin in patients with cancer [113,118].

In this systematic review, we also identified study protocols for upcoming/ongoing RCTs addressing various types of cancer. Moreover, assessing efficacy and safety, two of these protocols also aim to assess health-related quality of life [42,46]. On the other hand, we identified RCTs that aim to study the potential use of known antineoplastic agents against other non-oncological conditions. One finished study and two protocols investigate the potential use of imatinib, a BCR-ABL tyrosine kinase inhibitor, in patients with severe coronavirus disease 2019 (COVID-19) [47,48,50], as various authors reported findings that the use of imatinib may be beneficial in hospitalized patients with COVID-19 [120,121]. However, an RCT identified in this systematic review showed that imatinib did not reduce the time to discontinuation of ventilation and supplemental oxygen in hospitalized COVID-19 patients who required supplemental oxygen [47]; this RCT was considered multimorbidity. We also identified an RCT protocol aiming to investigate the potential use of leuprolide, a gonadotropin-releasing hormone analogue, in women with Alzheimer’s disease [49].

This systematic review aimed to identify the most recent RCTs that focused on drug repurposing in oncology. A limitation of this study, commonly found in literature reviews, regards the identification of potentially eligible studies. For example, we used only MEDLINE and not other databases. However, MEDLINE covers most of the studies worldwide and assures a collection of high-quality papers. In addition, the search algorithm is available and published in this article to enhance transparency—an algorithm that exhaustively searches for all potential uses in oncology. This algorithm allowed us to also identify the repurposing of antineoplastic agents for other non-oncological conditions. Furthermore, we chose to include study protocols separately in order to study ongoing/upcoming studies. The aim of this systematic review was to publish the “state of the art”; the high heterogeneity in methodologies, outcomes, indications, etc. did not make it possible to apply meta-analysis. Another limitation is the low number of RCTs that were included in the systematic review. A detailed description of all included studies (including a GRADE assessment regarding the quality of evidence) is available in the Supplementary Material.

## 5. Conclusions

This systematic review revealed that only a few clinical trials in drug repurposing in oncology were placebo-controlled or standard-of-care-alone-controlled. Metformin has been studied for potential use in various types of cancer, including prostate, lung, and pancreatic cancer. Other studies assessed the possible use of the antiparasitic agent mebendazole in colorectal cancer and of propranolol in multiple myeloma or, when combined with etodolac, in breast cancer. Overall response rate, overall survival, progression-free survival, disease-free survival, and the notification of drug-related side effects and adverse reactions were the most commonly used variables/outcomes. Among the most common limitations of the identified studies were the small sample size, the high clinical heterogeneity of the participants, and the baseline clinical characteristics. It is essential to conduct further trials with a higher number of participants and a more homogeneous study population considering the stage of the neoplastic disease and the baseline clinical profile, especially multimorbidity, concurrent medication, mental health, and functional status. The age of the participants is an important factor, but it may also depend on the length of time a concurrent disease has been present and whether it is well controlled or means a high morbidity burden for the patient. It is also vital to consider other factors, such as socio-economics and lifestyle, as they are key to an optimal person-centered approach and to obtaining the best results. By addressing all these aspects, data from randomized controlled clinical trials may have the potential to answer important clinical questions, and by using common protocols, we will be able to make plausible comparisons and conduct useful meta-analyses.

## Figures and Tables

**Table 1:** Search strategy performed in MEDLINE.

Query	Search Algorithm	Number of Records ^ⴕ^
#1	(“Drug Repositioning”[MeSH Terms] OR “drug reposit*”[Title/Abstract] OR “drug repurpos*”[Title/Abstract] OR “drug resc*”[Title/Abstract] OR (“repurpos*”[Title/Abstract] AND “drug”[Title/Abstract]) OR (“reposit*”[Title/Abstract] AND “drug”[Title/Abstract]) OR “new indication”[Title/Abstract] OR “indication change”[Title/Abstract] OR “change indication”[Title/Abstract] OR “another indication”[Title/Abstract])	13,876
#2	(“antineoplastic agents”[MeSH Terms] OR “neoplasms”[MeSH Terms] OR “medical oncology”[MeSH Terms] OR “surgical oncology”[MeSH Terms] OR “carcinoma”[MeSH Terms] OR “hodgkin disease”[MeSH Terms] OR “leukemia, lymphocytic, chronic, b cell”[MeSH Terms] OR “lymphoma”[MeSH Terms] OR “myelodysplastic syndromes”[MeSH Terms] OR “tumo*”[Title/Abstract] OR “neoplas*”[Title/Abstract] OR “cancer”[Title/Abstract] OR “cancer*”[Title/Abstract] OR “malignan*”[Title/Abstract] OR “oncolog*”[Title/Abstract] OR “carcinom*”[Title/Abstract] OR “epitheliom*”[Title/Abstract] OR “Hodgkin”[Title/Abstract] OR “lymphom*”[Title/Abstract] OR “leukemia”[Title/Abstract] OR “Leucocythaemia”[Title/Abstract] OR “Leucocythemia”[Title/Abstract] OR “Leukocythemia”[Title/Abstract] OR “sarcoma”[Title/Abstract] OR “Reticulolymphosarcoma”[Title/Abstract] OR “Germinoblastoma”[Title/Abstract] OR “blastoma”[Title/Abstract] OR “myelodyspl*”[Title/Abstract] OR “Dysmyelopoietic”[Title/Abstract] OR “Anticancer”[Title/Abstract] OR “antineoplast*”[Title/Abstract] OR “antitumo*”[Title/Abstract] OR “chemotherap*”[Title/Abstract])	5,170,828
#1 AND #2		4370

^ⴕ^ Literature search performed on 20 November 2022.

## Supplementary Materials

The following supporting information can be downloaded at: https://www.mdpi.com/article/10.3390/cancers15112972/s1. Table S1: A general overview of the randomized controlled clinical trials included in the systematic review and GRADE assessment; File S1. PRISMA checklist: PRISMA 2020 main checklist and abstract checklist.
